# Effects of Ketamine on Rodent Fear Memory

**DOI:** 10.3390/ijms21197173

**Published:** 2020-09-28

**Authors:** Kwang H. Choi, Rina Y. Berman, Michael Zhang, Haley F. Spencer, Kennett D. Radford

**Affiliations:** 1 Department of Psychiatry, Uniformed Services University, Bethesda, MD 20814, USA; kwang.choi@usuhs.edu (K.H.C.); rina.berman.ctr@usuhs.edu (R.Y.B.); michael.zhang.ctr@usuhs.edu (M.Z.); 2 Center for the Study of Traumatic Stress, Uniformed Services University, Bethesda, MD 20814, USA; 3 Program in Neuroscience, Uniformed Services University, Bethesda, MD 20814, USA; haley.spencer@usuhs.edu; 4 Daniel K. Inouye Graduate School of Nursing, Uniformed Services University, Bethesda, MD 20814, USA

**Keywords:** ketamine, fear conditioning, fear memory, intravenous, stress, PTSD

## Abstract

Ketamine, a multimodal anesthetic drug, has become increasingly popular in the treatment of pain following traumatic injury as well as treatment-resistant major depressive disorders. However, the psychological impact of this dissociative medication on the development of stress-related disorders such as post-traumatic stress disorder (PTSD) remains controversial. To address these concerns, preclinical studies have investigated the effects of ketamine administration on fear memory and stress-related behaviors in laboratory animals. Despite a well-documented line of research examining the effects of ketamine on fear memory, there is a lack of literature reviews on this important topic. Therefore, this review article summarizes the current preclinical literature on ketamine and fear memory with a particular emphasis on the route, dose, and timing of ketamine administration in rodent fear conditioning studies. Additionally, this review describes the molecular mechanisms by which ketamine may impact fear memory and stress-related behaviors. Overall, findings from previous studies are inconsistent in that fear memory may be increased, decreased, or unaltered following ketamine administration in rodents. These conflicting results can be explained by factors such as the route, dose, and timing of ketamine administration; the interaction between ketamine and stress; and individual variability in the rodent response to ketamine. This review also recommends that future preclinical studies utilize a clinically relevant route of administration and account for biological sex differences to improve translation between preclinical and clinical investigations.

## 1. Introduction

Ketamine has become a multi-purpose tool within the field of medicine for a variety of indications from trauma analgesia to a treatment for depression and post-traumatic stress disorder (PTSD). Due to the preservation of ventilation and favorable hemodynamic stability, ketamine has gained popularity among military and civilian first responders as the primary analgesic medication of choice to treat pain caused by traumatic injury [1] and is touted as a successful last-resort option for patients suffering from treatment-resistant depression. However, there are concerns regarding the potential impacts of ketamine on traumatic memory and adverse long-term psychological outcomes because of its dissociative and hallucinogenic properties, and questions remain regarding the neuropharmacological mechanisms by which ketamine exerts its downstream effects.

Due to the inherent moral dilemmas of conducting prospective clinical experiments involving traumatic events and stress-related disorders in humans, researchers often turn to preclinical rodent models to explore ketamine’s effects on fear memory and PTSD-like behaviors. However, results from preclinical research are often contradictory due to a variety of confounders. Despite a well-documented line of research examining ketamine’s effects on rodent fear memory, there is a lack of published reviews on this topic. Therefore, this review aims to describe the most recent preclinical literature that has explored the effects of peri-trauma ketamine administration on rodent fear memory and PTSD-like behaviors with consideration of the route, dose, and timing of ketamine administration along with additional variables that may affect ketamine effects on fear memory. Furthermore, this review summarizes the potential biological mechanisms by which ketamine may interfere with fear memory in rodents and suggests recommendations for future preclinical studies that will enhance clinical translation.

Rodent fear memory is comprised of multiple phases including acquisition, extinction, and recall. Fear memory acquisition can occur through various methods, but the most common is through Pavlovian fear conditioning, where a neutral stimulus (sound tone) is paired with an aversive stimulus (footshock) such that a future exposure to the neutral stimulus produces freezing behavior. Humans undergo the same phenomenon in response to a traumatic event is often associated with sights, sounds, or smells present at the time of trauma. Fear extinction refers to a process whereby rodents slowly reduce freezing behaviors when repeatedly exposed to a non-reinforced neutral stimulus (sound tone with no footshock). Essentially, a rodent is creating a new, safe memory that overwrites the previous fear memory. The concept of fear extinction underlies the process of cognitive behavioral therapy in humans, whereby a patient will describe or discuss a traumatic event in repeated sessions. Lastly, fear recall occurs after a rodent has successfully demonstrated fear extinction and is re-exposed to the original environment where fear memory acquisition took place. The neutral stimulus and/or the original fear conditioning environment alone can trigger freezing behavior. Similarly, humans can experience this same phenomenon when visiting the scene of a previous traumatic event or experiencing a sight, sound, or smell previously associated with the trauma.

For this review, we utilized the following inclusion criteria: (1) rodent studies including mice or rat species; (2) subanesthetic racemic (*R,S*) ketamine doses; (3) the utilization of a rodent fear conditioning paradigm; (4) ketamine’s effects on fear memory acquisition, fear memory extinction, and fear recall; and (5) male or female sex. We utilized the following exclusion criteria: (1) human or non-rodent studies; (2) anesthetic ketamine doses; and (3) chronic ketamine administration. We included studies only with subanesthetic ketamine doses because these are clinically relevant doses that are recommended for trauma analgesia without severe dissociation [2]. Although we included both male and female sexes in our search, interestingly, almost all the preclinical studies utilized only male rodents. We excluded studies with chronic ketamine administration because this type of prolonged exposure paradigm is not clinically relevant with regards to acute ketamine administration following trauma. Moreover, chronic ketamine administration may produce different biological effects as compared to acute or sub-chronic ketamine administration.

## 2. Effects of Ketamine on Fear Memory

We observed a distinct pattern among the preclinical literature that may account for the key differences in observed behavioral outcomes across ketamine studies on fear memory as shown in Table 1. This pattern generally falls under the umbrella of ketamine administration, individual differences, and stress exposure. In the following section, we review the relevant literature utilizing different routes, doses, and timing of ketamine administration as a guiding framework, and discuss individual differences and stress exposure as additional confounding variables.

### 2.1. Route of Ketamine Administration

The vast majority of preclinical researchers utilize an intraperitoneal (IP) route to administer ketamine to rodents, despite the intravenous (IV) route being the preferred route of ketamine administration in patients. The differences in route of administration can lead to conflicting results, as evidenced by IP and IV ketamine producing opposite effects within the same experimental paradigm. A 10 mg/kg IP injection of ketamine facilitated the extinction of fear memory, while a 10 mg/kg (2 h) IV infusion delayed fear extinction in rats [22]. These results highlight the importance of the route of ketamine administration (IV vs. IP) and the duration of the drug peak effects. Differences in the pharmacokinetics of IP and IV administration could potentially influence ketamine’s effect on fear memory, as an IV infusion allows the drug plasma concentration to maintain a steady state over a longer duration following a lower dose in the body, compared to an IP method that produces a rapid peak and then trough of plasma concentration [23,24]. Additionally, IV ketamine does not undergo first-pass metabolism because it bypasses the liver upon initial administration, leading to greater bioavailability at target organs. On the other hand, IP ketamine undergoes hepatic metabolism after administration, which can lead to increased metabolite production and reduced initial ketamine availability compared to that of a similar IV dose. Furthermore, the results of IV administration depend on whether the drug is administered through a rapid bolus (one-time IV injection) or a prolonged infusion with a mechanical syringe pump. Consistent with these observations, a preclinical study observed that a ketamine bolus (2 and 5 mg/kg, IV) produced rapid and robust dissociative stereotypy [24], while a ketamine infusion (10 mg/kg, IV) over 2 h produced hypo-locomotor activity (a sign of sedation and analgesia) in rats [20,22]. Because the IV route is rarely used in preclinical studies, it is critical to utilize the IV ketamine administration technique to a larger extent in preclinical research in order to better model the clinically relevant route of ketamine administration. Additionally, comparing the bolus and infusion effects of ketamine on fear memory in rodents would provide valuable feedback for optimal treatment in a clinical setting.

### 2.2. Ketamine Dosage

In addition to the route of administration, the dose of ketamine administered also plays a crucial role in the differential effects of ketamine observed on fear memory. Many studies have reported dose-dependent effects of ketamine on fear memory. A dose of ketamine at 10 mg/kg is frequently observed within the preclinical literature and is often cited as an effective antidepressant dose in rodents [25]. In rats, 10 mg/kg, but not 3 mg/kg, IP ketamine increased escape-type fear and decreased avoidance-type fear [21]. Rats that received ketamine (10 mg/kg, IP) after the reactivation of contextual fear had less freezing the following day compared to those that received 20 mg/kg, indicating that a lower ketamine dose impaired the reconsolidation of contextual fear memory [11]. Radford and colleagues found a similar result when ketamine infused at varying doses (2, 10, and 20 mg/kg, IV, 2 h) after fear conditioning produced an inverted-U-shaped dose–response curve, such that the 10 mg/kg dose produced a greater enhancement of fear memory than the low and high doses in rats [22]. The high-dose ketamine infusion (20 mg/kg, IV, 2 h) increased dissociative behavior and hyper-locomotion, while the medium-dose ketamine infusion (10 mg/kg, IV, 2 h) produced hypo-locomotion and sedative-like effects [22]. It is possible that dissociative behaviors and hyper-locomotion interfere with fear memory consolidation, while mild sedation enhances memory consolidation. Clinically, these findings suggest that peri-trauma ketamine administered for analgesia may have dose-dependent psychological effects that could impact the development of stress-related disorders.

### 2.3. Timing of Ketamine Administration

Researchers utilize the timing of ketamine administration in fear conditioning studies to simulate different treatment models. For example, administering ketamine before fear conditioning represents a prophylactic treatment; administration immediately after fear conditioning mimics trauma analgesia; and a delayed dose after fear conditioning or prior to extinction training simulates a therapeutic modality. Ketamine exerts differential effects depending on when it is administered in relation to the exposure to fear conditioning or extinction training—in other words, timing matters. The sections below include a review of preclinical results from ketamine administered before fear conditioning, immediately after fear conditioning, and at later time points after fear conditioning (including administration before fear extinction testing).

#### 2.3.1. Pre-Fear Conditioning

There are a limited number of studies that have examined the effects of ketamine administered prior to fear conditioning on fear memory behaviors. These types of models are generally referred to as prophylactic in that the researchers evaluate the protective effects of ketamine on fear memory if administered prior to a trauma. Most studies vary with regards to the ketamine dose (10 to 30 mg/kg, IP) and the amount of time between ketamine administration and fear conditioning (30 min to 1 month), along with the time until the fear memory assessments (1 to 11 days). For example, ketamine administration (10 mg/kg, IP) 15 min before contextual fear conditioning in rats decreased fear memory when measured the next day [8]. Clifton et al. (2018) administered ketamine (25 mg/kg, IP) 30 min before footshock fear conditioning, and rats demonstrated substantially less conditioned contextual freezing 2 days later, suggesting ketamine may prevent memory consolidation [6]. However, others have administered ketamine (10 and 30 mg/kg, IP) 60 min prior to fear conditioning and found no effects on freezing behavior in extinction trials conducted 1 to 11 days later [12,14,17]. It is possible that a high dose of ketamine administered within a short time prior to fear conditioning with the foot-shock paradigm may reduce conditioning effects due to the analgesic effects of ketamine and thus by dampening fear memory consolidation in the animals. Ketamine administered 1 week prior to fear conditioning showed mixed results with reports of either no effects [17] or decreases [14] in subsequent freezing behavior. Interestingly, McGowan et al. (2017) also reported that prophylactic ketamine administered 1 month and 1 h before fear conditioning had no effect on freezing behaviors, suggesting a specific time window of 1 week prior to fear conditioning for optimal prophylaxis in mice [14]. Overall, there are mixed results regarding the effects of prophylactic ketamine injections on fear behaviors. These conflicting results may stem from different ketamine doses, fear conditioning paradigms, species, and time points for fear memory testing. Overall, prophylactic ketamine studies are difficult to translate clinically because one cannot predict when a traumatic experience will occur for any specific individual.

#### 2.3.2. Immediately Post-Fear Conditioning

The effects of subanesthetic ketamine doses administered in the immediate period following fear conditioning on short-term freezing behavior are mixed. Generally, the premise is that post-trauma ketamine may serve to either enhance or disrupt memory consolidation and thus impact fearful memory formation and the development of stress-related disorders. A pair of investigations administered ketamine at a higher dose range (25–30 mg/kg, IP) immediately following foot-shock fear conditioning and found no effects on freezing behavior when it was measured 24–48 h later [6,17]. In contrast to studies that utilized IP injections, Radford et al. (2018) administered a ketamine infusion (10 mg/kg, IV, 2 h) after foot-shock fear conditioning and reported impaired fear extinction at 2 and 3 days post-fear conditioning as well as enhanced fear renewal at 4 days post-conditioning in rats [22]. This suggests that a prolonged and slow IV ketamine infusion may have differential effects on fear memory consolidation compared to short-term exposure by IP injection.

In addition to the effects on short-term memory, several investigations have tested the effects of ketamine administered immediately after fear conditioning on long-term fear memory. For instance, Kulyk et al. (2017) administered ketamine (10 mg/kg, IP) immediately after fear conditioning and found reduced freezing during initial extinction learning 10 days later, but no overall effect on freezing behavior during fear extinction recall testing 11 days later in rats [12]. Similarly, McGowan (2017) administered a higher dose of ketamine (30 mg/kg, IP) 1 h after fear conditioning and reported no effects on fear expression in mice 4 days later [14]. Lastly, Radford et al. (2020) administered a ketamine infusion (10 mg/kg, IV, 2 h) following fear conditioning and found no effects on long-term cued fear renewal when measured 7 days post-fear conditioning in rats [20]. These data suggest that regardless of the route of administration, ketamine administered immediately after fear conditioning has little impact on long-term fear memory, suggesting that ketamine administered in the post-trauma period may not pose significant long-term risks for stress-related disorders, including delayed-onset PTSD.

#### 2.3.3. Delayed Post-Fear Conditioning

Preclinical researchers also delay ketamine administration after fear conditioning to mimic the use of ketamine as a pharmacological treatment for stress-related disorders. Delayed ketamine treatment models typically occur at 24 h post-fear conditioning or in the immediate period (30–60 min) prior to fear extinction testing. Girgenti et al. (2017) reported decreased freezing on the second day of extinction testing following low-dose ketamine (10 mg/kg, IP) administration 24 h after fear conditioning in rats [13]. This suggests a narrow time window for ketamine’s effects. However, Asim et al. (2020) reported decreased fear generalization up to 2 weeks when higher-dose ketamine (30 mg/kg, IP) was administered 22 h after fear conditioning in mice [17]. In contrast to decreased fear behaviors, Radford et al. (2018) reported impaired fear extinction and enhanced fear renewal when a low-dose ketamine (10 mg/kg, IV, 2 h) infusion was administered 24 h after fear conditioning in rats [22]. These data suggest that the effects of delayed ketamine administration on fear memory may depend on the dosage and duration of ketamine exposure.

In addition to delayed ketamine models, researchers have administered ketamine prior to fear extinction testing to test the potential therapeutic effects on stress-related disorders such as PTSD. Higher ketamine doses (25–30 mg/kg, IP) administered 30–60 min prior to fear extinction testing had no effects on freezing behaviors when they were tested at 2 days [6] or 4 days [14] after fear conditioning. However, pre-extinction ketamine (8 and 25 mg/kg, IP) did enhance the fear recall measured 2 days after extinction in rats [6], suggesting delayed ketamine effects. Interestingly, lower-dose ketamine (10 mg/kg, IP) given 1 h pre-extinction enhanced the early extinction learning measured 10 days later [12]. These results show that ketamine administration prior to fear extinction testing produces variable effects depending on the ketamine doses and the timing of extinction testing after fear conditioning.

### 2.4. Ketamine and Stress

The experience of stress plays a crucial role in the development of psychiatric disorders, and is one of the diagnostic criteria for PTSD. Indeed, disruption of fear extinction can occur when rodents are exposed to various types of stressors such as the forced swim test, forced restraint, and maternal separation [26,27,28]. However, the combination of different types of stressors and fear learning introduces another variable that may produce differential effects for ketamine when compared to the models that use unstressed animals. Hence, understanding the interaction between ketamine and stress, and its implications for fear memory has significant clinical value. While many studies have used unstressed rats, some studies have utilized stress models to investigate ketamine’s effects on fear. In a chronic social defeat stress (CSDS) model of depression, mice exposed to 10-day CSDS before contextual fear conditioning exhibited a reduction of freezing compared to non-stressed controls, and ketamine administration partially restored freezing in depressed mice [7]. By contrast, neither prophylactic ketamine nor two weeks of CSDS impacted freezing in a contextual fear conditioning test in mice [29]. This negative finding could be attributed to the 4-week delay between ketamine administration and fear conditioning testing [29] compared to a 5-day delay [7]. In a predator-scent stress (PSS) model of PTSD, rats exposed to PSS and then treated with ketamine 1 h after stress for three days exhibited a significant increase in freezing behavior when re-exposed to the scent cue 30 days later, suggesting ketamine may enhance fear memory during a stressed state [3]. In addition, a chronic stress model using chronic corticosterone (CORT) for 21 days increased freezing during long-term extinction retention, which was reversed by the pre-extinction administration of ketamine (15 mg/kg, IP) in rats [12]. Studies that utilize a combination of stress exposure and fear conditioning along with ketamine administration translate well to the clinical environment since most individuals experience traumatic stress at the time of injury and then receive analgesic doses of ketamine. Therefore, these results provide important information about the interactions between ketamine and stress regarding fear memory, and further research is necessary.

### 2.5. Individual Variability

Individual variability is an important factor in determining the susceptibility or resilience to the development of certain stress-related disorders, as not all individuals exposed to a traumatic event develop PTSD. Consequently, researchers can expect individual differences among animals, and if correctly identified, these factors may predict individual susceptibility or resilience to PTSD. For example, a ketamine infusion (10 mg/kg, IV, 2 h) immediately after cued fear conditioning did not enhance long-term fear memory when rats were tested 1 week later [20]. However, a significant positive correlation was noted between ketamine-induced CORT levels and long-term fear memory in individual animals [20]. Therefore, animals that are more sensitive to the effects of ketamine on hypothalamic–pituitary–adrenal (HPA) axis stimulation may experience greater long-term fear memory. On a similar note, Saur et al. (2017) classified individual rats as “extreme behavioral responders” (EBR) or “minimal behavioral responders” (MBR) according to the level of freezing on a situational reminder (SR) test 1 week after fear conditioning. The EBR rats that received ketamine showed heightened freezing on the SR test compared to the MBR rats [5]. Overall, the issue of individual variability is complex and difficult to study, as it requires a larger number of animals for statistical power and more in-depth behavioral characterization, along with the identification of biological markers. However, understanding an individual’s resilience and/or susceptibility to any given disease has profound clinical implications for not only predicting at-risk individuals but also determining the optimal pharmacological treatments for those patients. As ketamine gains traction in the treatment of various psychiatric disorders, preclinical researchers should continue to explore the role of individual variability with regard to potential ketamine interactions in and effects on laboratory animals.

## 3. Molecular Mechanisms

**Figure 1:**
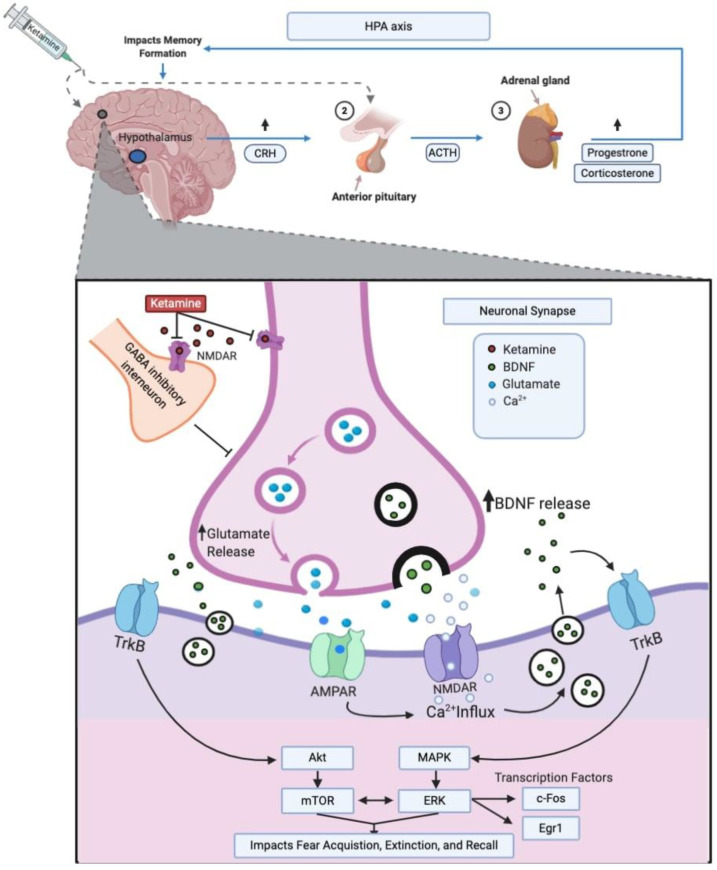
Summary of ketamine’s proposed molecular mechanisms. CRH, corticotrophin-releasing hormone; ACTH, adrenocorticotropic hormone; HPA, hypothalamic–pituitary–adrenal; GABA, gamma-aminobutyric acid; AMPAR, alpha-amino-3-hydroxy-5-methyl-4-isoxazolepropionic acid receptor; NMDA, *N*-methyl-D-aspartate receptor; TrkB, tropomyosin receptor kinase B; BDNF, brain-derived neurotrophic factor; AKT, protein kinase B; mTOR, mammalian target of rapamycin; MAPK, mitogen-activated protein kinase; ERK, extracellular signal-regulated kinase; Egr1, early growth response 1; Ca^2+^, calcium. This illustration was created using Biorender.com.

An increasing number of preclinical investigations aim to describe the molecular systems involved in the effects of ketamine on fear memory, as shown in Table 1. Ketamine primarily acts on glutamatergic *N*-methyl-d-aspartate (NMDA) receptors but is relatively non-selective and, as such, modulates an array of other molecular systems [30,31]. Those molecular systems include the brain-derived neurotrophic factor (BDNF), mammalian target of rapamycin (mTOR), and extracellular signal-regulated kinase (ERK) pathways and the Fos and Egr1 transcription factors. A number of brain regions, most prominently the hippocampus, amygdala, and prefrontal cortex (PFC), are implicated in the effects of ketamine on fear memory. Furthermore, ketamine stimulates stress hormone secretion, which may play a role in fear memory consolidation. These molecular systems and their role in ketamine’s effects on fear memory are outlined in the following sections and summarized in Figure 1.

### 3.1. Glutamatergic Signaling

The *N*-methyl-D-aspartate (NMDA) and α-amino-3-hydroxy-5-methyl-4-isoxazolepropionic acid (AMPA) receptors are two glutamatergic receptors that are central to learning, memory, and plasticity [32,33], with significant involvement in fear memory [34,35] and in ketamine’s mechanism of action [36]. A proposed mechanism by which ketamine acts on NMDA receptors (NMDAR) is the disinhibition hypothesis, whereby ketamine selectively blocks NMDAR on GABAergic neurons to disinhibit glutamate release [36,37]. Ketamine (10 mg/kg) administered subcutaneously (SC) before the trace fear conditioning paradigm, in which the tone and footshock are separated by a small “trace” time interval, markedly reduced trace conditioned fear memory in mice, but had no effect in the standard delay conditioning where the tone co-terminates or is immediately followed by shock [18]; similar findings have been reported in rats [9]. This reduction in fear was abolished by NMDA receptor subtype 2B (GluN2B) knockdown in GABAergic somatostatin-expressing (SST) interneurons, which is consistent with the disinhibition hypothesis [18]. The route of administration may have contributed to these findings, as subcutaneous ketamine (10 mg/kg) decreased fear in mice, whereas an IV infusion at the same dose had fear-inducing effects in rats [22]. Ketamine (10 mg/kg, IP) administered before fear memory reactivation enhanced freezing 24 h later in rats; these effects were blocked by the infusion of ifenprodil, a GluN2B antagonist, into the basolateral amygdala (BLA) 15 min prior to ketamine injection, indicating GluN2B involvement in fear memory reconsolidation [4]. Mice exhibiting a depression-like phenotype as a result of CSDS had decreased hippocampal GluN2B levels and impaired contextual fear memory compared to non-depressed controls, which were partially restored by 5 mg/kg IP ketamine [7]. Fear-generalized mice had increased GluN2B protein expression in the infralimbic (IL)-PFC and basolateral amygdala (BLA), and injection of ketamine (30 mg/kg, IP) 22 h after fear conditioning alleviated fear generalization and decreased GluN2B protein expression in those regions [17]. Furthermore, the alleviation of fear generalization by ketamine was abolished by the infusion of ifenprodil into the IL-PFC, further indicating the role of GluN2B in fear generalization [17]. Although primarily an NMDAR antagonist, ketamine also acts on AMPAR to affect fear memory; administration of the AMPAR antagonist 2,3-dihydroxy-6-nitro-7-sulphamoyl-benzo(f)quinoxaline (NBQX) partially blocked facilitation of extinction by ketamine (10 mg/kg, IP) in rats, implicating AMPAR in extinction learning after ketamine administration [13]. By and large, preclinical studies report that ketamine decreases fear memory by inhibiting NMDAR, reducing GluN2B expression, and activating AMPAR; however, the route of ketamine administration and the nature of the fear paradigm used may contribute to the disparate findings.

### 3.2. BDNF

BDNF is a protein associated with learning and memory that is often implicated in the antidepressant effects of ketamine [36,38]. In ketamine’s mechanism of action, increased glutamate release activates postsynaptic AMPAR, which depolarizes the cell and prompts the vesicular release of BDNF, which binds to the tropomyosin receptor kinase B (TrkB) receptor to affect further downstream pathways, namely, ERK and mTOR [38,39]. Fear generalization in mice resulted in reduced BDNF protein expression in the BLA and PFC, and ketamine (30 mg/kg, IP) administration at 22 h post-fear conditioning alleviated fear generalization and increased BDNF protein expression in those regions [17]. The intra-medial PFC (mPFC) infusion of the TrkB inhibitor ANA-12 decreased BDNF protein expression and blocked ketamine’s abolishment of fear generalization, indicating that BDNF is implicated in decreased fear generalization following ketamine administration [17]. Ketamine (10 mg/kg, IP) given after the reactivation of contextual fear memory decreased fear memory reconsolidation while increasing BDNF mRNA levels in the prelimbic cortex in rats, indicating that upregulated BDNF diminishes fear reconsolidation [11]. By contrast, ketamine administration (10 mg/kg, IP) three days before a BDNF assay did not alter BDNF protein levels in the hippocampus or frontal cortex of rats, although the animals exhibited increased freezing behaviors one day after ketamine administration [5]. Moreover, although no behavioral outcome was measured, a ketamine infusion at an analgesic dose (10 mg/kg, IV, 2 h) increased BDNF protein in the amygdala of rats; this effect was dose-dependent and not seen with a 40 mg/kg infusion [40]. The inconsistent effects of ketamine administration on BDNF levels in the brain may be due to the timing of the BDNF assay, because BDNF levels may fluctuate following ketamine administration [41]. Overall, studies reporting decreased fear memory after ketamine implicated increased BDNF in this effect, but it is worth noting that changes in BDNF are dose- and time-dependent.

### 3.3. mTOR and ERK

Another mechanism of ketamine’s action includes the mTOR and ERK pathways. The extracellular signal-regulated kinase (ERK) and the protein kinase B (AKT) pathways are upstream modulators of the mTOR pathway [13,42] and are implicated in fear memory [43,44]. The mammalian target of rapamycin (mTOR) pathway is involved in cell differentiation, learning, and plasticity [45] and is implicated in neuronal growth and morphology [46]. Ketamine dose-dependently activated mTOR, ERK, and AKT; the mTOR antagonist rapamycin blocked the antidepressant effects of ketamine, indicating functional significance of the mTOR pathway in mood and anxiety disorders [46]. Ketamine (10 mg/kg, IP) administered 24 h after fear conditioning facilitated fear extinction in rats on the second of three subsequent extinction days and increased p-ERK and p-AKT in the mPFC [13]. An intra-mPFC administration of rapamycin blocked mTOR and inhibited ketamine’s enhancement of fear extinction [13]. A study using an IV ketamine infusion (10 and 40 mg/kg, 2 h) reported dose-dependent effects on p-ERK levels in the mPFC and hippocampus, brain regions involved in fear memory, of rats [40]. The ketamine-induced upregulation of mTOR and ERK decreased fear memory in rats; however, alterations of these proteins vary with the dose and route of ketamine administration. Furthermore, while mTOR is manipulated by antagonism with rapamycin, further study is required to assess the functional role of ERK in the effects of ketamine on fear memory.

### 3.4. Transcription Factors

Through interaction with the aforementioned molecular pathways, ketamine also exerts influence on many transcription factors such as c-Fos, ΔFosB, and Egr1. A descriptive study reported dose-dependent increases in c-Fos levels in the mPFC and amygdala, which are important limbic regions associated with fear memory, in rats following IV ketamine infusion (10 and 40 mg/kg, 2 h) [40]. Immunohistochemistry analysis revealed that c-Fos^+^-positive cells were increased in the hippocampus as a result of prophylactic ketamine (30 mg/kg, IP) administered one week prior to contextual fear conditioning in mice, coinciding with reduced fear memory [16]. c-Fos levels were increased in the mPFC of rats when measured two days after ketamine (10 mg/kg, IP) administration, coinciding with facilitated extinction memory [13]. ΔFosB, a splice variant of FosB, is implicated in stress susceptibility and resilience [47]. Prophylactic ketamine (30 mg/kg, IP) administered one week before contextual fear conditioning reduced freezing and increased ΔFosB levels in the hippocampus of mice, which were nullified by the viral injection of ΔJunD, an antagonist of ΔFosB transcription [16]. Early growth response 1 (Egr1), also known as zif268, was upregulated in rats during fear reconsolidation, and its effects were abolished by the intrahippocampal infusion of Egr1 antisense DNA [48]. A study showed that ketamine administration (10 mg/kg, IP) after the reactivation of fear memory impaired reconsolidation and downregulated Egr1 mRNA in the hippocampus of rats [11]. Taken together, these results imply that the Fos and Egr1 transcription factors are altered after ketamine administration concurrent with a decrease in fear, but further study is necessary to validate the functional significance of those transcription factors, particularly Egr1, on fear memory in laboratory animals following ketamine administration.

### 3.5. Brain Glucose Metabolism

There is a lack of in vivo brain imaging studies using ketamine administration in rodents. Brain imaging, such as fluorodeoxyglucose-positron emission tomography (FDG-PET), can reveal regional differences in brain energy utilization in living animals. For instance, ^18^F-FDG-MicroPET imaging two days after ketamine administration (10 mg/kg, IP) in fear-conditioned rats revealed no change in glucose metabolism in the hippocampus, frontal cortex, or amygdala [5]. This lack of effect of ketamine on regional glucose metabolism may be attributed to the delayed imaging after ketamine administration (e.g., 48 h delay between ketamine administration and FDG-PET imaging). By contrast, an ^18^F-FDG-PET scan taken immediately after an IV ketamine infusion (10 mg/kg, 2 h) revealed robust effects on regional glucose metabolism in rats [22]. The ketamine infusion increased glucose metabolism in the hippocampus, amygdala, and hypothalamus, key regions associated with fear memory and stress, while decreasing metabolism in the cerebellum [22]. One of the major advantages of in vivo brain imaging is the capability for repeated measures in the same animals, which allows the investigation of the time course of glucose metabolism following ketamine administration. With the improved sensitivity and resolution of PET scans, it is possible to determine key brain regions affected by ketamine administration in laboratory animals. Based on the differences found between the previous studies, it would be important to consider the route of ketamine administration (IP bolus vs. IV infusion) and the time course of regional glucose metabolism following ketamine administration.

### 3.6. Stress Hormones and HPA Axis

Stress hormones released following trauma regulate limbic activity and may enhance or impair memory formation based on their concentrations and durations of exposure within the brain [49,50,51,52]. It is well established that subanesthetic doses of ketamine increase stress hormone levels in humans [53,54,55] as well as in animals [20,56]. For instance, an IV ketamine infusion (10 or 40 mg/kg, 2 h) transiently increased levels of plasma CORT in rats [56]. A follow-up study confirmed increased levels of CORT and progesterone, a precursor to CORT that is released under stress [57], following an IV ketamine infusion (10 mg/kg, 2 h) in rats [20]. However, another study reported that ketamine administration (0.5, 5, and 15 mg/kg, IP) did not alter serum CORT levels when they were measured 20 min later in rats [3]. Interestingly, the stress-induced elevation of CORT levels was reduced by ketamine administration in that study. This suggests that ketamine may have differential effects on HPA axis function depending on the absence or presence of stress exposure, as well as acute vs. chronic stress. Furthermore, the route of ketamine administration (IP bolus vs. IV infusion) may produce differential effects on stress hormone levels based on those studies [3,56]. Nevertheless, previous human studies consistently reported transient increases in cortisol levels following an IV ketamine infusion [53,54,55]. Overall, these studies suggest that IV ketamine infusion at subanesthetic doses increases stress hormone levels in both rodents and humans, which increases fear memory.

## 4. Conclusions

In summary, preclinical research on ketamine’s effects on fear memory has demonstrated inconsistent findings, with results showing increased, decreased, or no effect on fear memory in laboratory animals. However, there are multiple factors contributing to these differences, such as the inconsistent routes, doses, and timing of ketamine administration as well as stress exposure at the time of ketamine administration. Multiple brain regions and molecular pathways are implicated in the effects of ketamine on fear memory, although the findings in molecular systems are again dependent upon the route (bolus vs. infusion) and timing (pre- vs. post-fear conditioning) of ketamine administration in animals. Taken together, the route and the timing of ketamine administration are two variables that greatly impact the fear memory outcomes found within this review, and investigators should consider them when evaluating preclinical studies reporting ketamine’s effects on fear memory. With careful considerations of preclinical methodology, we can enhance our understanding of the behavioral and molecular mechanisms of ketamine’s effects on fear and stress-related disorders and improve clinical translation from the bench to bedside.

## 5. Future Directions

Research teams have explored many variables within a variety of fear memory and fear extinction ketamine studies; however, the effects of sex and route of drug administration are often neglected. Male rodents were predominantly used in the ketamine preclinical literature for this review (Table 1), which leads to a scarcity of literature on sex differences when describing ketamine’s effects on stress and fear memory. A recent study administered prophylactic (*R,S*)-ketamine (30 mg/kg, IP) and its metabolites (*2S,6S*)-hydroxynorketamine (HNK) and (*2R,6R*)-HNK (0.025-30 mg/kg, IP) one week prior to contextual fear conditioning in mice; racemic ketamine and (*2S,6S*)-HNK attenuated learned fear in male mice but not in female mice [19]. Since both men and women experience traumatic injury or other traumatic events, utilizing both sexes in ketamine research with regards to fear memory is of utmost importance. Additionally, studies typically employ IP injections of ketamine in rodents due to convenience and simplicity, despite IV ketamine being a more clinically relevant route of administration. The different routes of administration can have opposing effects on fear memory, despite the same fear conditioning paradigm and ketamine dose being used in rats [22]. Therefore, to maximize clinical translation and relevance, preclinical researchers should consider the addition of sex differences and route of administration in their study design.

## Figures and Tables

**Table 1:** Summary of literature on the effects of ketamine and fear memory in rodents.

Study	Sex, Species, Strain, Age	Dose, Route, Timing	Behavioral Results	Molecular Results
**Ketamine Increased Fear Memory**				
Juven-Wetzler et al., 2014 [3]	Male, rats, S-D	0.5, 5, 15 mg/kg IP 1 h after predator-scent stress	Increased fear memory	Attenuated increase in CORT after predator-scent stress exposure
Honsberger et al., 2015 [4]	Male, rats, S-D, adult	10 mg/kg IP, 15 min before or just after reactivation	Pre-reactivation ketamine increased fear memory	Effect nullified by GluN2B antagonist ifenprodil
Saur et al., 2017 [5]	Male, rats, Wistar, 12 w	10 mg/kg IP 1 day before situational reminder	Increased fear memory	No change in brain glucose metabolism or BDNF levels
Clifton et al., 2018 [6]	Male, rats, Lister Hooded, adult	8, 25 mg/kg IP 30 min before or just after CFC and extinction	Pre-extinction ketamine impaired fear extinction consolidation	-
Yang et al., 2018 [7]	Male, mice, C57BL/6 J, 8 w	5 mg/kg IP 1 d before CFC	Increased contextual fear memory	Partially restored hippocampal GluN2B subunit density, LTP induction, NMDAR EPSC amplitude in depressed mice
**Ketamine Decreased Fear Memory**				
Calzavara et al., 2009 [8]	Male, rats, Wistar, 5 months	10 mg/kg IP 15 min before CFC	Reduced fear memory	-
Bolton et al., 2012 [9]	Male, rats, S-D, adult	8 mg/kg SC 30 min before trace or delay FC	Reduced trace fear memory No effect on delay fear memory	Increased GABA_B1b_ in amygdala of trace conditioned animals receiving ketamine
Ito et al., 2015 [10]	Male, mice, 129SvEv/C57BL/6N, P60–75	10 mg/kg IP after observational fear	Prevented increased passive avoidance fear memory	Increased AMPAR/NMDAR current ratio, and reduced silent synapses in IL-PFC–BLA pathway
Duclot et al., 2016 [11]	Male, rats, S-D, 8 w	10, 20 mg/kg IP after reactivation	Impaired reconsolidation	10 mg/kg decreased Egr1 mRNA, increased BDNF mRNA
Kulyk et al., 2017 [12]	Male, rats, Long-Evans, adult	10 mg/kg IP 60 min pre-FC or pre-extinction, just after FC 15 mg/kg IP 60 min pre-extinction	Post-FC and pre-extinction 10 mg/kg ketamine decreased fear recall 15 mg/kg nullified CORT-induced increase in fear memory	
Girgenti et al., 2017 [13]	Male, rats, S-D, 7–9 w	10 mg/kg IP 24 h after FC	Enhanced extinction and reduced return of fear memory	Increased p-ERK and p-AKT Effect nullified by mTOR antagonist rapamycin
McGowan et al., 2017 [14]	Male, mice, 129S6/SvEvTac, 8 w	30 mg/kg IP 1 week, 1 month, 1 day, 1 h before CFC	Ketamine 1 week before CFC reduced fear memory	-
McGowan et al., 2018 [15]	Male, mice, 129S6/SvEvTac, 7 w	30 mg/kg IP 1 w before CFC	Reduced fear memory	Altered nucleotide and amino-acid-derived neurotransmitter metabolism
Mastrodonato et al., 2018 [16]	Male, mice, 129S6/SvEvTac, ArcCreER^T2^ x ChR2–EYFP	30 mg/kg IP 1 w before CFC	Reduced fear memory	Increased ΔFosB levels; ΔFosB transcription antagonist blocked ketamine effect
Asim et al., 2019 [17]	Male, mice, C57BL/6, 6–8 w	30 mg/kg IP 1 h or 1 week before, just after, 22 h after FC	Ketamine 22 h after FC reduced fear generalization	Increased BDNF and lowered GluN2B protein expression in BLA and IL-PFC; BDNF and GluN2B antagonists restored fear generalization
Ali et al., 2020 [18]	Male, mice, C57BL/6J, 10 w	10 mg/kg SC 15 min before trace and delay FC	Decreased trace fear memory No effect in delay fear memory	GluN2B knockdown in somatostatin-expressing (SST) interneurons abolished ketamine reduction of trace fear memory
Chen et al., 2020 [19]	Male and female, mice, 129S6/SvEv, 7 w	30 mg/kg IP 1 w before CFC	Attenuated contextual fear memory in males	Blocked AMPAR current bursting
**No Effects of Ketamine on Fear Memory**				
Radford et al., 2020 [20]	Male, rats, S-D	10 mg/kg IV after FC	No effect on long-term fear memory	Increased CORT and progesterone
**Mixed Effects of Ketamine on Fear Memory**				
Babar et al., 2001 [21]	Male, rats, Wistar	3 mg/kg IP 15 min before testing 10 mg/kg IP 15 min before testing	3 mg/kg decreased freezing 10 mg/kg increased escape, decreased avoidance and freezing	-
Radford et al., 2018 [22]	Male, rats, S-D	2, 10, 20 mg/kg IV after FC 10 mg/kg IP after FC	10 mg/kg IV increased fear memory 10 mg/kg IP decreased fear memory	Infusion after FC increased glucose metabolism in amygdala, hippocampus, hypothalamus, midbrain; decreased in cerebellum

IV, intravenous; IP, intraperitoneal; SC, subcutaneous; S-D, Sprague-Dawley; FC, fear conditioning; CFC, contextual fear conditioning; CSDS, chronic social defeat stress; LTP, long-term potentiation; mPFC, medial prefrontal cortex; BLA, basolateral amygdala; ILC, infralimbic cortex; PLC, prelimbic cortex; BDNF, brain-derived neurotrophic factor; p-ERK, phosphorylated extracellular signal-regulated kinase; p-AKT, phosphorylated AKT; EPSC, excitatory postsynaptic current; Egr1, early growth response 1; CORT, corticosterone.

## Disclaimer

The opinions and assertions expressed herein are those of the authors and do not necessarily reflect the official policy or position of the Uniformed Services University (USU) or the Department of Defense. This work was prepared by military and civilian employees of the U.S. Government as part of the individuals’ official duties and, therefore, is in the public domain and does not possess copyright protection (public domain information may be freely distributed and copied; however, as a courtesy, it is requested that USU and the author be given appropriate acknowledgement).
